# Elevated plasma level of neutrophil gelatinase-associated lipocalin (NGAL) in patients with breast cancer

**DOI:** 10.7150/ijms.58789

**Published:** 2021-05-13

**Authors:** Ching-Ting Wei, I-Ting Tsai, Cheng-Ching Wu, Wei-Chin Hung, Chin-Feng Hsuan, Teng-Hung Yu, Chia-Chang Hsu, Jer-Yiing Houng, Fu-Mei Chung, Yau-Jiunn Lee, Yung-Chuan Lu

**Affiliations:** 1 Division of General Surgery, Department of Surgery, E-Da Hospital, Kaohsiung, 82445 Taiwan.; 2 Department of Emergency, E-Da Hospital, Kaohsiung, 82445 Taiwan.; 3 Division of Cardiology, Department of Internal Medicine, E-Da Hospital, Kaohsiung, 82445 Taiwan.; 4 Division of Gastroenterology and Hepatology, Department of Internal Medicine, E-Da Hospital, Kaohsiung, 82445 Taiwan.; 5 Division of Endocrinology and Metabolism, Department of Internal Medicine, E-Da Hospital, Kaohsiung, 82445 Taiwan.; 6 School of Medicine, College of Medicine, I-Shou University, Kaohsiung, 82445 Taiwan.; 7 The School of Chinese Medicine for Post Baccalaureate, College of Medicine, I-Shou University, Kaohsiung, 82445 Taiwan.; 8 School of Medicine for International Students, College of Medicine, I-Shou University, Kaohsiung, 82445 Taiwan.; 9 Department of Nutrition, College of Medicine, I-Shou University, Kaohsiung, 82445 Taiwan.; 10 Department of Chemical Engineering, I-Shou University, Kaohsiung, 82445 Taiwan.; 11 Department of Biomedical Engineering, I-Shou University, Kaohsiung, 82445 Taiwan.; 12 Department of Electrical Engineering, I-Shou University, Kaohsiung, 82445 Taiwan.; 13 Division of Cardiology, Department of Internal Medicine, E-Da Dachang Hospital, Kaohsiung, 80794 Taiwan.; 14 Health Examination Center, E-Da Dachang Hospital, Kaohsiung, 80794 Taiwan.; 15 Lee's Endocrinology Clinic, Pingtung, 90000 Taiwan.

**Keywords:** neutrophil gelatinase‑associated lipocalin, breast cancer, white blood cell count, monocyte count, neutrophil count, lymphocyte count, platelet count

## Abstract

**Background:** Neutrophil gelatinase‑associated lipocalin (NGAL), also known as lipocalin 2, siderocalin, 24p3 or uterocalin, plays a key role in inflammation and in different types of cancer. In this study, we investigated whether plasma NGAL levels were altered in patients with breast cancer. The relationship between plasma NGAL levels and pretreatment hematologic profile was also explored.

**Methods:** Plasma NGAL concentrations were measured using ELISA in breast cancer patients and control subjects. A total of 75 patients with breast cancer and 65 age- and body mass index-matched control subjects were studied. All of the study subjects were female.

**Results:** Plasma NGAL level was found to be elevated in the patients with breast cancer compared to the control subjects (94.3 ng/mL (interquartile range 39.3-207.6) vs. 55.0 ng/mL (interquartile range 25.8-124.7), p = 0.007). Multiple logistic regression analysis revealed that NGAL was independently associated with breast cancer, even after adjusting for known biomarkers. Furthermore, NGAL level was elevated in the breast cancer patients who were negative progesterone receptor status, had a histologic grade ≥ 2, clinical stage III, and pathologic stage T2+T3+T4. In addition, NGAL level was significantly correlated with white blood cell (WBC) count, monocyte count, neutrophil count, and platelet count (all p < 0.01). Moreover, WBC count, neutrophil count, monocyte count, lymphocyte count, platelet count, and NGAL level gradually increased as the stage progressed.

**Conclusions:** Increased plasma NGAL levels were associated with breast cancer independently of risk factors, and were correlated with inflammatory biomarkers. These results suggest that NGAL may act through inflammatory reactions to play an important role in the pathogenesis of breast cancer.

## Introduction

Breast cancer is one of the most common cancers in women worldwide. Well-known risk factors for breast cancer include a high body mass index (BMI), increased alcohol consumption, sedentary lifestyle, hormone replacement therapy for menopause, exposure to radiation, early age at menarche, gene mutations, and metabolic disorders. In addition, inflammatory cytokines may play an important role 1. Chronic inflammation has been causally associated with various types of cancer 1-3, and numerous studies have reported that inflammatory infiltrate may be a strong risk factor for cancer development in chronic inflammatory conditions. Lipocalin proteins are involved in inflammation and detoxification processes caused by immune system activation in mammals, and functional roles of lipocalins in human cancers have been determined 4.

Neutrophil gelatinase‑associated lipocalin (NGAL), also known as lipocalin 2, siderocalin, 24p3 or uterocalin, is overexpressed in many pathologic conditions, including cancer. NGAL is a 25-kDa glycoprotein that was originally identified as a partner in a covalent complex with neutrophil gelatinase, also known as matrix metalloproteinase 9 (MMP-9) 5,6. It is frequently associated with tumor stage, size, and invasiveness, and experimental results have demonstrated that NGAL has multiple functions in various cancers, including stimulating proliferation 7, inhibiting apoptosis 8, and promoting the epithelial-to-mesenchymal transition 9. In addition, the NGAL gene in humans is more highly expressed in luminal epithelial cells than in myoepithelial cells 10, and most breast carcinomas are thought to develop from luminal epithelial cells. Hence, NGAL may actively participate in breast cancer progression 9. NGAL levels have also been strongly correlated with lymph node metastasis, poor histologic grade, high carcinoma proliferation ability and poor prognosis in breast cancer patients 11.

However, data regarding the role of NGAL and the association between NGAL and hematologic profile in patients with breast cancer are relatively limited. Therefore, the aim of this study was to investigate the role of NGAL in breast cancer. To this end, we measured pretreatment plasma NGAL levels and pretreatment hematologic profiles in a Chinese population with breast cancer.

## Methods

### Study participants

We enrolled 140 Chinese females in this study, including 75 with breast cancer and 65 healthy controls. The breast cancer patients (n = 75) were newly diagnosed and had histologically confirmed breast cancer with no prior surgical, chemotherapy, or radiotherapy treatment for breast cancer, and who attended E-Da Hospital between January 2020 and November 2020. The healthy control subjects (n = 65) were matched for age and BMI with the breast cancer group. For all subjects, comprehensive questionnaires were used to collect medical information. Complete history was obtained including lifestyle behaviors, medical history, menstrual and reproductive history, and menopausal status, as well as a family history of breast cancer and other cancers. Because the questionnaires were completed prior to undergoing surgery or radio/chemotherapy, any influence of treatment was unlikely. All of the patients and controls who participated in this study were fully informed of the aims of the study and gave written informed consent for their participation. The study protocol was approved by the Human Research Ethics Committee at E-Da Hospital.

### Anthropometric measurements and clinical examinations

Physical and clinical examinations were performed and anthropometric measurements were recorded for all of the participants. Height (to the nearest 0.5 cm) and weight (to the nearest 0.1 kg) were measured. BMI was calculated as weight in kg divided by the square of height in meters. Seated blood pressure was also measured by a trained nurse with a digital automatic blood pressure monitor (Omron model HEM-907, Omron, Japan) after the patients had rested for 5 minutes. All of the control subjects were confirmed to be free from benign or malignant breast diseases through physical examinations and mammography. Women with a history or family history of any tumor or cancer were excluded. The diagnosis of breast cancer was confirmed histologically in each case, and estrogen receptor status was determined. The staging of breast cancer was determined according to the TNM system.

### Laboratory measurements

Plasma biochemical parameters were measured after overnight fasting including triglycerides, total cholesterol, creatinine, and glucose, which were measured using standard commercial methods on a parallel, multichannel analyzer (Hitachi7170A, Tokyo, Japan) as in our recent report 12. Peripheral complete blood cell count was determined using an automated cell counter (XE-2100 Hematology Alpha Transportation System, Sysmex Corporation, Kobe, Japan). To minimize the confounding effect of infection, subjects with a white blood cell (WBC) count below 4.0 × 10^9^/l or greater than 10.0 × 10^9^/l were re-checked and studied extensively to rule out the existence of chronic infections on the basis of physicians interview, physical examination, and urinalysis.

### Plasma NGAL measurements

All blood samples were drawn after overnight fasting and plasma samples were kept at -80 °C for subsequent assay. The concentrations of plasma NGAL were determined using commercial enzyme immunoassay kits (Cloud-Clone Corp., Katy, USA). The intraassay coefficient of variation was <10% for NGAL. ELISA was performed according to the instructions of the manufacturer, who stated that the assay had excellent specificity for the detection of human NGAL, and no significant cross-reactivity or interference with analogues was observed. Samples were measured in duplicate in a single experiment.

### Statistical analysis

Data normality was analyzed using the Kolmogorov-Smirnov test. Continuous, normally distributed variables are presented as mean ± SD, and non-normally distributed variables as median (interquartile range [IQR]). Statistical differences in the variables were compared using unpaired Student's *t*-tests or Wilcoxon rank sum test (as indicated) for continuous variables. One-way analysis of variance was performed to examine the effect of each variable among the tumor stage groups. Since the distributions of serum triglycerides and plasma NGAL were skewed, logarithmically transformed values were used for the statistical analysis. These variables were assessed for independent associations with the presence of breast cancer in multiple logistic regression analysis using the control subjects as the reference category. Spearman rank correlation coefficients were used to examine correlations between plasma NGAL and the values of other parameters. All of the statistical analyses were two-sided, and a p value < 0.05 was considered to be significant. All analyses were performed using SAS statistical software, version 8.2 (SAS Institute Inc., Cary, NC, USA).

## Results

Among 106 consecutive breast cancer patients, 31 were excluded due to the following reasons: twenty-one patients refused to join the study after surgery, and 10 patients could not be provided complete information regarding demographic history, biochemical data, and medication treatment. The final study population included 75 patients.

### Clinical and biochemical characteristics of study subjects

Table 1 shows the baseline clinical and biochemical data of the study participants with and without breast cancer. Compared to the healthy control subjects, plasma levels of NGAL were significantly higher among those with breast cancer (55.0 ng/ml (IQR: 25.8-124.7) versus 94.3 ng/ml (IQR: 39.3-207.6, p = 0.007). Furthermore, the patients with breast cancer had higher fasting glucose, WBC count, neutrophil count, monocyte count, and platelet count than the healthy control subjects. The mean age, BMI, blood pressure, total cholesterol, triglycerides, serum glutamate oxaloacetate transaminase, serum glutamic pyruvic transaminase, creatinine, estimated glomerular filtration rate, albumin, lymphocyte, red blood cells, hemoglobin, and hematocrit were similar in both groups.

### Associations between plasma NGAL and breast cancer

Multivariate logistic regression analysis was performed to estimate the effects of plasma NGAL level together with other breast cancer risk factors on the presence of breast cancer. The results showed that the presence of breast cancer was associated with BMI, fasting glucose, neutrophil count, and NGAL level (Table 2).

### Association between NGAL level and clinicopathologic markers

NGAL level was elevated in the breast cancer patients who were progesterone receptor (PR) negative, had a histologic grade ≥ 2, clinical stage III, and pathologic stage T2+T3+T4. However, there were no significant associations between estrogen receptor (ER) status (positive: negative), HER-2 receptor status (positive: negative), tumor size (≤ 1: > 1), and lymph node metastasis (N0+N1: N2+N3) and plasma NGAL level (all p > 0.05).

### Correlations among NGAL level and pretreatment hematologic parameters

**Figure 1:**
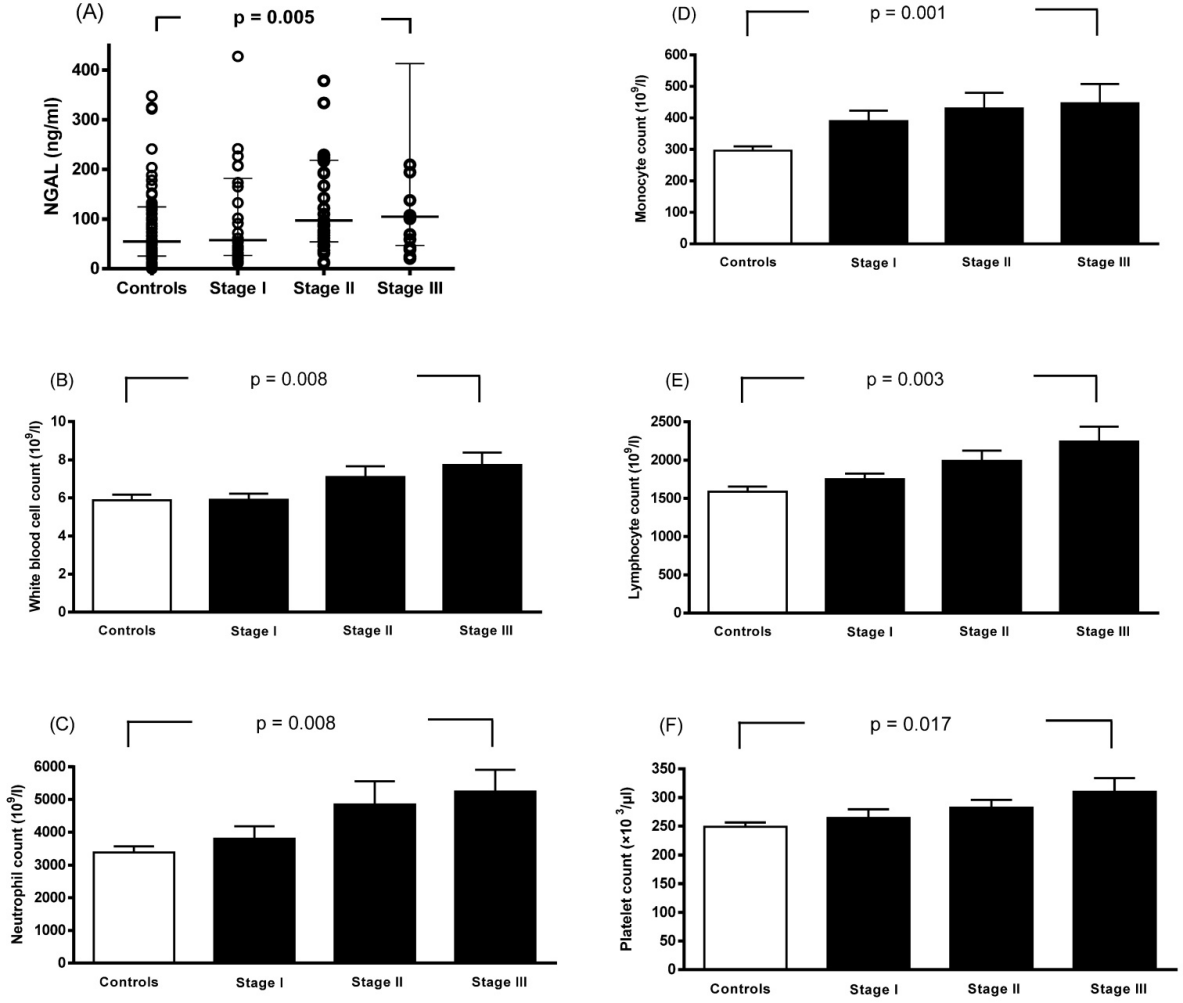
Associations between neutrophil gelatinase-associated lipocalin (A), white blood cell count (B), neutrophil count (C), monocyte count (D), lymphocyte count (E), platelet count (F), and stage progression of breast cancer. Bars represent the mean ± SD or median (interquartile range). Differences between groups were analyzed using one-way analysis of variance.

Spearman rank correlation analysis showed that plasma NGAL levels were correlated with WBC count, monocyte count, neutrophil count, and platelet count (Table 4). Linear contrast analysis was conducted to evaluate the correlations among WBC count, neutrophil count, monocyte count, lymphocyte count, platelet count, NGAL level, and tumor stage (Figure 1). The results showed that WBC count, neutrophil count, monocyte count, lymphocyte count, platelet count, and NGAL levels gradually increased as the stage progressed (p < 0.01).

## Discussion

In this study, we demonstrated that plasma NGAL concentrations were significantly elevated in a fully adjusted model in patients with breast cancer. Furthermore, the level of NGAL gradually increased as the stage progressed, along with elevations in PR-negative status, histologic grade ≥ 2, clinical stage III, and pathologic T2+T3+T4. Moreover, we found significant correlations between plasma NGAL concentrations and WBC count, monocyte count, neutrophil count, and platelet count. To the best of our knowledge, this is the first report to describe elevated NGAL plasma concentrations in patients with breast cancer.

Several molecular forms of NGAL have been identified: a disulfide-linked homodimer (46-kDa), a monomer (25-kDa), and a disulfide-linked heterodimer with human neutrophil gelatinase B (135-kDa) 6,13. NGAL is expressed in many other types of cells besides neutrophils. When NGAL is present in a complex with MMP-9, there is less degradation of MMP-9 due to the effects on NGAL on the stability of MMP-9. This results in higher gelatinolytic activity of MMP-9 on the extracellular matrix 13,14. This biological function of NGAL may influence the development of diverse cancer types 14-17. NGAL is an acute-phase protein that has been implicated in diverse physiological processes in mice, including ion transport, apoptosis, inflammation, cell survival, and tumorigenesis 18,19. NGAL has been shown to promote leukocyte recruitment in the tumor microenvironment through ion-mediated chemokine production 20 and also in activated immune cells such as monocytes and neutrophils 6,21, and the dimer has been shown to be the major molecular form of free NGAL secreted by neutrophils 22. As NGAL can activate leukocytes, it can be regarded as a pro-inflammatory 20, immunomodulating 23, and apoptosis-inducing cytokine 24. In the present study, plasma NGAL levels were found to be elevated in the patients with breast cancer, and elevated plasma NGAL levels were associated with total WBC count, monocyte count, and neutrophil count. Based on the findings of previous studies and our data, we propose that NGAL should be considered as a marker of inflammation that participates in the process of breast cancer.

Chronic inflammatory reactions have been shown in tumors, and they may be associated with cancer progression and chemoresistance. NGAL gets part of its name from neutrophils, as it was determined to be released by neutrophils at sites of infection and inflammation 23. A previous study found that the expression of NGAL could be up-regulated by interleukin-1beta in a type II pneumocyte-derived cell line through the induction of the nuclear factor-kappaB (NF-kB) pathway 25. Furthermore, NGAL has been shown to be strongly induced by NF-kB in thyroid carcinomas, an important factor involved both in tumor growth and in the link between chronic inflammation and neoplastic development 26,27. Hypoxic tumors, defined as tumor growth occurring at a distant region from blood vessels in tumor tissue, have been implicated in tumor metastasis, tumor invasion, and chemotherapy resistance, and are thus considered to be an indicator of tumor malignancy 28-30. Nakamura et al. demonstrated that NGAL was a useful plasma marker for hypoxic tumors 31. Furthermore, previous studies have identified that NGAL can actively promote breast cancer metastasis by inducing the production of vascular endothelial growth factor, angiogenesis, epithelial-to-mesenchymal transition and cell migration, and invasion through multiple signaling pathways, including phosphoinositide-3-kinase/protein kinase B/NF-κB and hypoxia inducible factor-1α/extracellular signal-regulated kinase 10,32-34. Notably, silencing NGAL in breast cancer cells has been shown to reduce tumor progression. Moreover, a few studies have correlated the expression of NGAL to the clinical outcomes of cancer patients 11,35,36. Bauer et al. 11 showed that in primary human breast cancer, NGAL expression was a predictor of poor prognosis, and suggested that the presence of NGAL may assist in risk assessment and identifying a subset of patients requiring more aggressive adjuvant therapy. Hu et al. 35 provided strong evidence for the role of NGAL in aggressive subtypes of breast cancer, metastasis and poor prognosis. Due to its property as a secretory protein, NGAL can be easily detected in the urine or blood circulation, and previous studies have suggested that NGAL may be a prognostic biomarker and noninvasive diagnostic test for breast cancer progression. Wenners et al. 36 also showed that in low-risk subgroups, NGAL was a predictive marker for a pathological complete response after neoadjuvant chemotherapy. Furthermore, NGAL was shown to be an independent prognostic factor for a decrease in disease-free survival in primary human breast cancer. In addition, Venkatesha et al. 37 showed that NGAL represses ras-induced expression of vascular endothelial growth factor in 4T1 cells through the down-regulation of ras mitogen-activated protein kinase and ras phosphatidylinositol-3-kinase signaling. Hence, this study provided new insights into the action of NGAL and raise the possibility that the administration of NGAL may be useful for inhibiting tumor angiogenesis, in addition to suppressing tumor metastasis, in cancers which show ras activation. In the present study, we found that NGAL was associated with breast cancer, and that its concentration gradually increased as the stage progressed in accordance with previous reports 10,11. In addition, our results also showed that NGAL concentration was positively correlated with platelet count. A previous study reported that a high platelet count was associated with metastasis and poor prognosis in cancer patients 38,39. Therefore, the correlation between NGAL and platelet count may explain the increased risk of breast cancer in patients with a high NGAL level.

Stuckey et al. showed that in normal human mammary epithelial cells, the expression of NGAL is controlled by estrogen 40, while in malignant human mammary epithelial cells, NGAL appears to escape from hormonal regulation, since this protein is most abundant in ER-negative breast cancer cell lines and primary tumor samples 11. Furthermore, Stoesz et al. found that in human breast tumors, the expression of NGAL was not significantly associated with HER-2/neu activation. In contrast, they found significant associations between NGAL expression and several other poor prognostic markers in patients with breast cancer, including ER- and PR-negative status and high proliferation (S-phase fraction) 41. Interestingly, in the present study, we demonstrated that NGAL was elevated in the patients with PR-negative status, histologic grade ≥ 2, clinical stage III, and pathologic T2+T3+T4. Hence, our findings suggest that NGAL may be actively contributive to breast cancer progression. However, NGAL was not elevated in the ER-negative patients in the present study, possibly due to the limited sample size.

Some limitations of this study need to be considered. First, our study population was relatively small, and so further studies with larger populations are needed. Moreover, the cross-sectional design limited our ability to infer any causal relationship between NGAL and breast cancer. A prospective cohort study is required to completely elucidate the importance of NGAL as a biomarker of breast cancer and the causative association between breast cancer and the changes in NGAL levels. In addition, although we controlled for other major cancer risk factors, the existence of unrecognized confounding variables is always possible. Further investigation is needed to investigate the association between NGAL plasma levels with the prognosis and survival of the breast cancer.

## Conclusions

In conclusion, we found that NGAL plasma concentrations were elevated in patients with breast cancer in our Chinese study population, and that there was possibly a close relationship between NGAL and inflammation and the development of breast cancer. NGAL may be involved in the complex interactions involving inflammatory or immune responses. Further investigations are required to explore the detailed mechanisms of NGAL in the development and progression of breast cancer, and its utility in breast cancer therapy.

## Figures and Tables

**Table 1:** Baseline clinical and biochemical characteristics of the study population

Parameter	Breast cancer	Healthy controls	*p*-value
No.	75	65	
Age (years)	55.3±11.2	56.0±7.1	0.702
Body mass index (kg/m^2^)	25.5±4.0	24.3±4.4	0.103
Systolic blood pressure (mmHg)	131±18	125±18	0.062
Diastolic blood pressure (mmHg)	77±10	77±13	0.702
Fasting glucose (mg/dl)	127.9±48.9	99.8±21.6	0.0001
Total cholesterol (mg/dl)	189.8±33.6	192.8±39.5	0.752
Triglycerides (mg/dl)	136(69-214)	88(66-122)	0.177
SGOT (U/L)	28.9±19.4	29.3±9.6	0.910
SGPT (U/L)	26.8±22.8	28.1±15.9	0.704
Creatinine (mg/dl)	0.95±0.18	0.93±0.09	0.459
Estimated GFR (ml/min/1.73 m^2^)	93.7±19.2	93.1±12.8	0.839
Albumin (g/dl)	4.3±0.3	4.2±0.3	0.586
White blood cell count (10^9^/l)	6.808±2.758	5.904±1.694	0.029
Neutrophil count (10^9^/l)	4488±2653	3389±1333	0.006
Monocyte count (10^9^/l)	426±212	296±101	<0.0001
Lymphocyte count (10^9^/l)	1790±559	1993±639	0.069
Red blood cells (×10^6^/μl)	4.52±0.53	4.42±0.32	0.199
Hemoglobin (g/dL)	12.6±1.7	12.8±1.0	0.379
Hematocrit (%)	38.1±5.3	38.6±2.5	0.475
Platelet count (×10^3^/μl)	280.6±82.9	249.4±54.9	0.014
NGAL (ng/ml)	94.3(39.3-207.6)	55.0 (25.8-124.7)	0.007

Data are mean ± SD or median (interquartile range). SGOT, serum glutamate oxaloacetate transaminase; SGPT, serum glutamic pyruvic transaminase; GFR, glomerular filtration rate, NGAL, neutrophil gelatinase-associated lipocalin.

**Table 2:** Multiple logistic regression analysis with the presence of breast cancer as the dependent variable

	exp(B)	95% confidence interval	*p*-value
Age	0.87	0.73-1.03	0.105
Body mass index	1.38	1.04-1.84	0.028
SBP	1.08	0.99-1.18	0.090
DBP	0.84	0.72-0.98	0.052
Total cholesterol	1.01	0.98-1.05	0.496
Triglycerides	1.00	0.99-1.02	0.898
Fasting glucose	1.07	1.01-1.12	0.012
SGOT	1.20	0.99-1.47	0.068
SGPT	0.90	0.78-1.03	0.121
Estimated GFR	0.96	0.88-1.05	0.404
White blood cell count	0.67	0.36-1.25	0.205
Neutrophil count	1.19	1.01-1.39	0.033
Monocyte count	2.04	0.84-4.93	0.113
Platelet count	1.02	0.99-1.03	0.079
NGAL	1.01	1.00-1.02	0.040

SBP, systolic blood pressure; DBP, diastolic blood pressure; SGOT, serum glutamate oxaloacetate transaminase; SGPT, serum glutamic pyruvic transaminase; GFR, glomerular filtration rate; NGAL, neutrophil gelatinase-associated lipocalin.

**Table 3:** Median (interquartile range) baseline plasma concentration of neutrophil gelatinase-associated lipocalin grouped according to clinicopathologic marker status

Parameters	N	NGAL (ng/ml)	*p*-value
**Estrogen receptor status**			
Positive	62	72.6 (27.2-137.9)	0.198
Negative	13	97.2 (42.6-213.2)	
**Progesterone receptor status**			
Positive	53	64.5 (33.3-115.9)	0.016
Negative	22	104.8 (45.5-226.2)	
**HER-2 receptor status**			
Positive	60	100.6 (44.2-213.2)	0.070
Negative	15	72.2 (26.9-122.1)	
**Histologic grade**			
1	25	61.4 (31.1-101.8)	0.006
≥2	50	132.9 (44.5-228.3)	
**Tumor size (cm)**			
≤1	17	72.2 (29.6±166.1)	0.271
>1	58	100.6 (43.0±217.0)	
**Clinical stage**			
Stage I	28	57.4 (26.8±182.0)	0.048
Stage II	31	97.4 (54.0-218.4)	
Stage III	16	104.7 (47.0-413.6)	
**Pathologic T stage**			
T0+T1	35	81.0 (31.0-168.7)	0.029
T2+T3+T4	40	99.1 (54.0-227.9)	
**Lymph node metastasis**			
N0+N1	69	90.4 (41.3-207.2)	0.149
N2+N3	6	151.1 (23.5-536.9)	

Data are median (interquartile range). NGAL, neutrophil gelatinase-associated lipocalin. Comparisons were performed using unpaired *t* tests and one-way analysis of variance (ANOVA).

**Table 4:** Spearman rank correlation coefficients of the study variables in the patients

	WBC count	Monocyte count	Neutrophil count	Lymphocyte count	Albumin	Red blood cells	Hemoglobin	Hematocrit	Platelet count	NGAL
WBC count	1	0.594**	0.939**	0.615**	-0.083	0.258**	0.135	0.106	0.343**	0.307**
Monocyte count		1	0.541**	0.233**	-0.036	0.131	0.187*	0.144	0.277**	0.257**
Neutrophil count			1	0.400**	-0.024	0.282**	0.121	0.114	0.403**	0.357**
Lymphocyte count				1	-0.136	0.130	0.246**	0.199*	0.234*	0.122
Albumin					1	0.318*	0.314*	0.291*	0.188	0.233
Red blood cells						1	0.391**	0.499**	0.279**	0.146
Hemoglobin							1	0.919**	0.080	-0.049
Hematocrit								1	0.174*	-0.021
Platelet count									1	0.296**
NGAL										1

NGAL, neutrophil gelatinase-associated lipocalin; WBC, white blood cell. *p < 0.05. **p < 0.01.
